# Judging the quality of evidence in reviews of prognostic factor research: adapting the GRADE framework

**DOI:** 10.1186/2046-4053-2-71

**Published:** 2013-09-05

**Authors:** Anna Huguet, Jill A Hayden, Jennifer Stinson, Patrick J McGrath, Christine T Chambers, Michelle E Tougas, Lori Wozney

**Affiliations:** 1 Centre for Pediatric Pain Research, IWK Health Centre, 5850/5980 University Avenue, PO Box 9700, Halifax, Nova Scotia B3K 6R8, Canada; 2 Department of Community Health and Epidemiology, Dalhousie University, 5790 University Avenue, Halifax, Nova Scotia B3H 1V7, Canada; 3 Hospital for Sick Children, Lawrence S Bloomberg Faculty of Nursing, University of Toronto, 555 University Avenue, Toronto, Ontario M5G 1X8, Canada; 4 Department of Psychology and Neuroscience, Dalhousie University, PO Box 15000, Halifax, Nova Scotia B3H 4R2, Canada; 5 Department of Psychiatry, Dalhousie University, 5909 Veterans' Memorial Lane, 8th Floor, Abbie J. Lane Memorial Building, QEII Health Sciences Centre, Halifax, Nova Scotia B3H 2E2, Canada; 6 Capital District Health Authority, Research and Innovation, Centre for Clinical Research Building, 117-5790 University Avenue, Halifax, Nova Scotia B3H 1V7, Canada

**Keywords:** GRADE, Prognosis, Quality of evidence

## Abstract

**Background:**

Prognosis research aims to identify factors associated with the course of health conditions. It is often challenging to judge the overall quality of research evidence in systematic reviews about prognosis due to the nature of the primary studies. Standards aimed at improving the quality of primary studies on the prognosis of health conditions have been created, but these standards are often not adequately followed causing confusion about how to judge the evidence.

**Methods:**

This article presents a proposed adaptation of Grading of Recommendations Assessment, Development and Evaluation (GRADE), which was developed to rate the quality of evidence in intervention research, to judge the quality of prognostic evidence.

**Results:**

We propose modifications to the GRADE framework for use in prognosis research along with illustrative examples from an ongoing systematic review in the pediatric pain literature. We propose six factors that can decrease the quality of evidence (phase of investigation, study limitations, inconsistency, indirectness, imprecision, publication bias) and two factors that can increase it (moderate or large effect size, exposure-response gradient).

**Conclusions:**

We describe criteria for evaluating the potential impact of each of these factors on the quality of evidence when conducting a review including a narrative synthesis or a meta-analysis. These recommendations require further investigation and testing.

## Background

Prognosis research examines the progression of a health condition over time in order to identify risk and protective factors that can alter the likelihood of a future event during the course of such a condition [1]. The evidence about the progression of a health condition derived from prognosis research is crucial to make informed decisions about the process to identify individuals who are at risk for poor outcomes, to facilitate early intervention and guide the development of preventive interventions that target modifiable prognostic factors [2]. Synthesizing results from prognosis research for these potential uses is, however, often challenging as primary study results are often inconsistent and difficult to interpret [3]. Individual study differences may be due to, for example, small sample sizes, adjustments for different variables in the analyses, or consideration of different subsets within the same population [4-7]. Standards have been proposed to guide the design, procedures, analysis and reporting of this research in an attempt to minimize the variability and improve the quality of the primary studies [4-7]. However, these standards are often not followed causing confusion about the prognostic value of individual factors [8] and limiting research application [9]. Due to the important implications of this research for improving health outcomes, providing guidelines about how to judge the evidence of this widely varied research is necessary. This manuscript presents a system that can help reviewers judge the evidence derived from prognostic factor research [10]. We suggest that Grading of Recommendations Assessment, Development and Evaluation (GRADE) [11] can be adapted for the assessment of evidence derived from prognostic factor research.

### GRADE: a framework to guide the judgment about the quality of evidence in a systematic review

GRADE was first developed to provide methodological guidance in reviewing intervention research; specifically, how to rate the quality associated with estimated effects of an intervention on a specific outcome, and how to grade the strength of recommendations regarding the intervention as part of a guideline development process [12]. When making judgments about the quality of evidence, the GRADE approach considers five factors that can decrease our confidence in estimates of effects: (1) study design and limitations in study design, (2) inconsistency of results across studies, (3) indirectness of the evidence, (4) imprecision and (5) publication bias; and three factors that can increase our confidence in estimates of effects from observational studies: (1) large estimates of treatment, (2) a dose–response gradient and (3) plausible confounding that would increase confidence in an estimate. The GRADE framework, widely used by researchers working on reviews and guidelines, and groups providing recommendations for health care professionals such as NICE [13], has also been formally adapted for use in grading the quality of evidence and strength of recommendation for diagnostic research [14]. Recently, Goldsmith and colleagues have also proposed using GRADE as a framework for prognostic studies [15]. They used the framework and definitions of GRADE to rate the quality of evidence for prognostic studies evaluating cold hyperalgesia as a prognostic factor in whiplash-associated disorders. They described the general framework that was followed; however, they failed to address all of GRADE's factors or provide enough information to allow replication of their GRADEing process for prognostic studies. The following sections outline specific aspects of the modified GRADE framework (for example, how risk of bias in primary prognosis studies were assessed), the process used to modify the GRADE framework, and suggestions for how to apply the new adapted framework to systematic reviews where meta-analyses are lacking.

### Applying the GRADE framework to prognosis research

Consistent with the GRADE principles, when synthesizing the evidence from prognosis research it is important to estimate the effect of a factor on an outcome and also to report the level of confidence in these findings. Therefore, in a systematic review of prognostic factors it is recommended to assess the quality of evidence for each outcome of interest across studies. Our team has applied the GRADE framework and concepts to assess the quality of a body of evidence from prognosis studies into four quality categories (high, moderate, low, and very low) according to the traditional GRADE framework (Table 1). Along with our descriptions of how the GRADE framework can be adapted and used in this kind of research, we provide examples from a recent systematic review of prognostic studies conducted in the field of recurrent pain in children and adolescents (Table 2) (Huguet A, et al., in preparation) Since there are times when it is not appropriate or possible to conduct meta-analysis of prognostic evidence due to diversity within the studies included in the review or to poor methodological quality, or both, we also describe variations on how to implement the GRADE systematic review framework when conducting a narrative synthesis. We are not presenting a formalized guideline. Our recommendations are based on the discussions between the co-authors and our experience conducting systematic reviews of prognostic research on pain.

**Table 1:** **Definitions of the four quality categories according to the original Grading of Recommendations Assessment, Development and Evaluation (GRADE)**[16]**, applicable to the modified GRADE**


High quality	We are very confident that the true effect lies close to that of the estimate of the effect
Moderate quality	We are moderately confident in the effect estimate: the true effect is likely to be close to the estimate of the effect, but there is a possibility that it is substantially different
Low quality	Our confidence in the effect estimate is limited: the true effect may be substantially different from the estimate of the effect
Very low quality	We have very little confidence in the effect estimate: the true effect is likely to be substantially different from the estimate of effect

Reprinted from [16] with permission from Elsevier.

**Table 2:** Summary of a systematic review of prognostic studies in the field of recurrent pain in children and adolescents

**Recent systematic review**	**Description**
Huguet A, et al., in preparation	We have conducted a systematic review of the literature examining risk and protective factors for the onset and course of several chronic pain conditions in children and adolescents. The scope of this review is very broad because we have considered multiple etiological and prognostic factors combined with multiple outcomes.
	Through a search of PubMed, EMBASE, PsycInfo, CINAHL, and Web of Science from inception until February 2013, we included all manuscripts that meet the following criteria: studies were included if they were a cohort prospective or retrospective study with at least 3 months of follow-up quantitatively investigating through inferential statistics the prediction of the onset, persistence of chronic or recurrent pain conditions and pain-related disability from childhood or adolescence. Studies were excluded if they were non-English or the population had cognitive impairments or life threatening illnesses.
	Throughout this article we will provide examples of evidence of potential risk and protective factors associated with the course of headaches in children and adolescents in an attempt to provide a practical overview of the application of the GRADE for prognostic evidence.

### GRADE framework for prognosis

We think most of the factors taken into consideration by the GRADE framework to rate the quality of evidence from intervention research are conceptually applicable when applying the GRADE to judge the quality of evidence from prognostic research. However, our proposed assessment approach to determine how much each of these factors influence the quality of evidence is different from the approach originally suggested for intervention research. Table 3 compares the factors that may lead to rating down or up the quality of evidence from intervention research with the factors that may lead to rating down or up the quality of evidence from prognosis research. Notable differences when rating the quality of evidence from prognostic research include the following. (1) When judging the quality of prognostic evidence the study design is not considered since longitudinal research designs are the only acceptable ones that provide prognostic evidence (design characteristics are considered in the risk of bias assessment). (2) Using the GRADE framework to evaluate the quality of evidence from prognosis research should begin with the phase of investigation. (3) Plausible confounding does not need to be considered as an additional factor to rate up the quality of evidence. Two main reasons lead us to this decision. First, the potential effects of confounding in both intervention and prognosis research are not interpreted in the same direction. In intervention research, the assumption is that lack of control for confounding can inflate the reported effect sizes, so that the intervention appears to be more effective than it actually is when evidence is from studies that do not adequately control for confounding. This assumption is not made in prognostic research. It is often unclear how confounders alter effect sizes in prognostic studies. Second, plausible confounding is indirectly considered when assessing risk of bias. When assessing risk of bias, we are not evaluating how this will influence the strength of the effect; rather, we are evaluating the internal validity of the studies. As we describe below in more detail, when considering the potential impact of study limitations on the quality of evidence from prognostic research, the reviewers should consider downgrading the quality of evidence for methodological limitations when analyses are not adequately adjusted for confounders (which may be responsible for spurious or attenuated relations between the factor and the outcome).

**Table 3:** Factors that may increase and decrease the quality level of evidence

**Evidence about intervention**[16]	**Evidence about prognosis**
Factors that may decrease the quality	
1. Research design	1. Phase of investigation
2. Study limitations	2. Study limitations
3. Inconsistency	3. Inconsistency
4. Indirectness	4. Indirectness
5. Imprecision	5. Imprecision
6. Publication bias	6. Publication bias
Factors that may increase the quality	
1. Large effect size	1. Moderate or large effect size
2. Exposure-response gradient	2. Exposure-response gradient
3. Plausible confounding	

Next we describe the assessment approach for considering the potential effect of each of these factors on the quality of evidence.

### Phase of investigation

When evaluating the overall quality of evidence, we suggest that researchers consider the phase of investigation. A high level of evidence for prognosis is derived from a cohort study design that seeks to generate understanding of the underlying processes for the prognosis of a health condition, called a phase 3 explanatory study, or a cohort study design that seeks to confirm independent associations between the prognostic factor and the outcome, called phase 2 explanatory studies [4,7]. Prospective or retrospective cohort studies that test a fully developed hypothesis and conceptual framework without serious study limitations, and confirmatory studies without serious limitations constitute high-quality evidence on prognosis. These studies should be prioritized as primary studies. In emerging areas of research, there may be a lack of available primary studies that meet this criterion. In this instance, predictive modeling studies or explanatory studies conducted in the earlier phase of investigation to generate a hypothesis (phase 1 explanatory studies) may be included. These studies should be judged as providing weaker evidence. Therefore, we propose that the starting point for the quality level of the evidence should be based on phase of investigation (see Table 4). Table 5 illustrates an example of the effect of phase of investigation as a starting point for judging the quality of evidence.

**Table 4:** Guide to judge the quality of evidence for prognosis

**Phase of investigation**	**Quality of evidence**	**Downgrade if:**	**Upgrade if:**
Explanatory research aimed to understand prognostic pathways (phase 3 explanatory study) and explanatory research aimed to confirm independent associations between potential prognostic factor and the outcome (phase 2 explanatory study)	High	Study limitations:	Moderate or large effect:
		- Serious limitations when most evidence is from studies with moderate or unclear risk of bias for most bias domains	- For meta-analysis: pooled effect is moderate or large,
		- Very serious limitations when most evidence is from studies with high risk of bias for almost all bias domains	- For narrative summary: moderate or large similar effect is reported by most studies
		Inconsistency:	Exposure-gradient response
		Unexplained heterogeneity or variability in results across studies with differences of results not clinically meaningful. This may be supported by:	- For meta-analysis: gradient is present between analyses for factors measured at different doses
		- For meta-analysis: significant heterogeneity detected by test of heterogeneity and large I^2^ value.	- For narrative summary: possible gradient exists within and between primary studies
		- For narrative summary: variations in effect estimates across studies with points of effect on either side of the line of no effect, and confidence intervals showing minimal overlap	
		Indirectness:	
Outcome prediction research or explanatory research aimed to identify associations between potential prognostic factors and the outcome (phase 1 explanatory study)	Moderate	The study sample, the prognostic factor, and/or the outcome in the primary studies do not accurately reflect the review question	
		Imprecision:	
		- For meta-analysis: (1) insufficient sample size and (2) no precise estimate of the effect size in the meta-analysis: confidence interval is excessively wide and overlaps the value of no effect and contain values implying that the factor plays an important role in protecting or putting the individual at risk	
	Low	- For narrative summary: within-study imprecision: (1) sample size justification is not provided and there are less than 10 outcome events for each prognostic variable (for dichotomous outcomes) OR there are less than 100 cases reaching endpoint (for continuous outcomes), and (2) no precision in the estimation of the effect size within each primary study, AND	
	Very low		
		- Across study imprecision: there are few studies and small number of participants across studies.	
		Publication bias:	
		We recommend downgrading unless:	
		- The value of the risk/protective factor in predicting the outcome has been repetitively investigated, ideally by phase 2 and 3 studies	

**Table 5:** The effect of phase of investigation when judging quality of evidence

**Factor**	**Example**
Phase of investigation	During the selection process of our review for risk or protective factors of the course of “headache“, a common recurrent pain condition, we did not put any restriction on phase of investigation. We did this because, in the child pain prognosis literature, studies are rarely grounded in theoretical frameworks so there are rarely any phase 3 explanatory study published [17] and phase 2 exploratory studies are not very common. Therefore, our review majorly includes phase 1 exploratory studies that identify potential prognostic factors which are particularly vulnerable to type I errors (false positive results). Consequently, the quality of evidence from the studies selected in our review could not be initially rated high.

Following phase of investigation considerations, the evidence can be upgraded or downgraded according to the following additional criteria.

### Reasons for downgrading the quality of evidence

#### Study limitations

The findings derived from individual prognostic studies are often limited for their methodological shortcomings. There are several tools available for assessing methodological limitations [18-20]. We recommend using the Quality in Prognosis Studies (QUIPS) tool [19,20], which rates individual studies according to the potential risk of bias associated with six domains: (1) study participation, (2) study attrition, (3) prognostic factor measurement, (4) outcome measurement, (5) confounding measurement and account, and (6) analysis. This tool, designed for use in prognostic factor studies to comprehensively assess risk of bias based on epidemiological principles, has demonstrated acceptable reliability [20]. The level of risk of bias associated with each domain can be rated as 'low’, 'moderate’ and 'high’ based on the responses that reviewers give to each item.

We suggest that, when assessing the risk of bias of a prognostic factor across studies for a specific outcome, reviewers should rate the evidence as having: (1) no serious limitations when most evidence is from studies at low risk of bias for most of the bias domains; (2) serious limitations when most evidence is from studies at moderate or unclear risk of bias for most of the bias domains; or (3) very serious limitations when most information is from studies at high risk of bias with respect to almost all of the domains.

Table 6 illustrates an example. As is similar to what happens when GRADE is used for intervention research, the study limitations are outcome and factor specific [21]. Consequently, one study may be associated with higher risk of bias when referring to one outcome or to one prognostic factor than one referring to another outcome or factor of interest. We advise conducting subgroup analyses to explore the impact of studies with high risk of bias on specific domains and from the overall review (that is, serious or very serious limitations). Sensitivity analyses may be conducted to restrict the synthesis to studies with lower risk of bias. These steps will inform how the study limitations influence the size of the effect. If studies with 'serious limitations’ or 'very serious limitations’ are included in the body of evidence to be evaluated, specific justification in a footnote for the relevant tables is suggested.

**Table 6:** The effect of considering study limitations when judging quality of evidence

**Factor**	**Example**
Study limitations	When conducting our comprehensive systematic review of prognostic factors of headaches in children, we reported for example that the evidence of headache severity as a prognostic factor for persistence of headache had serious limitations. This evidence comes from three studies [22-24] and all of them have moderate risk of bias. The sources of potential bias in these studies were: not appropriately accounting for important potential confounders in the design and in the analysis (in [22-24]), not presenting sufficient data to assess adequacy of analysis (in [23,24]), selective reporting of results (in [23,24]), and not using any conceptual framework when building a model (in [22-24]). Moreover, we sometimes observed no description of inclusion/exclusion criteria (in [23]), no description of sample frame and recruitment (in [23,24]), and inadequate information about participation rate (in [22-24]).

#### Inconsistency

Inconsistency occurs when there is unexplained heterogeneity or variability in results across studies. When this happens, the quality of evidence decreases. Different approaches to assess inconsistency can be applied if the systematic review incorporates narrative synthesis or meta-analysis.

To evaluate whether inconsistency exists, we recommend that reviewers employing meta-analysis base their decisions on their judgment of whether a clinically meaningful difference exists between the point estimates and their confidence intervals of primary studies in the review context, as well as statistical parameters derived from their meta-analyses. Reviewers may consider downgrading the quality of evidence for inconsistency when they observe the following statistical parameters as long as these differences are clinically meaningful: (1) estimates of the effect of the prognostic factor on the outcome vary across studies with the points of effect on either side of the line of no effect and their confidence intervals show minimal or no overlap; (2) the statistical test for heterogeneity, which tests the null hypothesis that all studies in a meta-analysis have the same underlying magnitude effect, shows a low *P* value; and (3) the I^2^, which quantifies the proportion of the variation in point estimates due to true study variations in effect size from one study to the next, is substantial (that is, 50% or greater [25]).

Potential inconsistency related to differences in the magnitude of effects, as described above, should ideally be explored with *a priori* defined subgroup analyses. Differences in the population, the duration of follow-up, the outcome, the prognostic factor, or study methods across studies may explain differences. In this case, we propose to estimate separate effects accordingly. If, after running separate subgroup meta-analyses, this hypothesis is supported by the data, we suggest that reviewers consider presenting separate pooled estimates instead of estimating an overall combined effect.

If a meta-analysis is not conducted, we recommend that reviewers consider downgrading for inconsistency when estimates of the prognostic factor association with the outcome vary in direction (for example, some effects appear protective whereas others show risk) and the confidence intervals show no, or minimal overlap. See an example in Table 7.

**Table 7:** The effect of considering inconsistency when judging quality of evidence

**Factor**	**Example**
Inconsistency	Our narrative systematic review included three studies examining chronological age as a prognostic factor for persistence of headache [26-28]. While Larsson and Sund [27] found in a 1-year cohort study with 2,465 schoolchildren aged between 12 and 15 years that the risk of persistence of frequent headaches increases with age (odds ratio = 1.88, 95% confidence interval = 1.65 to 2.15), Kienbacher and colleagues [26] and Wang and colleagues [28] did not reach the same conclusion when running longer term follow-up studies with children and adolescents with a headache diagnosis. Kienbacher and colleagues [26] performed a follow-up study on 227 children and adolescents with migraine or tension-type headache. The average follow-up period was 6.6 years, with the age ranging from 5 to 8 years. Wang and colleagues [28] performed an 8-year follow-up study with adolescents with migraine. In both cases, age was reported not to be associated with persistence of headache. There is inconsistency of results across these three studies; however, neither of the two longer term cohort studies reported either means and a measure of variance or results from the statistical analyses to judge the level of overlap of the confidence intervals between the three studies. Duration of the follow-up, outcome and headache population were very different across studies. As far as the duration of the follow-up is concerned, despite the fact that the cohort study with short-term follow-up reported a significant risk estimate for an advanced age which was not found in cohort studies with longer term follow-ups, we did not find a plausible explanation for such potential influence of the duration of follow-up on the association between age and persistence of headaches. However, the outcome and the headache population could explain some of this heterogeneity [29]. Consequently, we did not consider the inconsistency across findings examining chronological age as a prognostic factor for headache persistence to be very serious and rated this item as serious inconsistency.

Inconsistency cannot be assessed when only a single study within the existing body of literature has estimated the effect. In these cases, this criterion may be considered as 'not applicable’. However, we still recommend the reviewers to downgrade the quality of evidence since this is an indicator that the literature is not well established in the area. If observed inconsistency is unexplained, reviewers should decide whether the inconsistency is serious or very serious and justify why they have made this decision with a footnote.

#### Indirectness

Indirectness exists when the participant population, prognostic factor(s) and/or outcomes considered by researchers in the primary studies do not fully represent the review question defined in the systematic review. The judged quality of evidence decreases because the results derived from the primary studies are less generalizable for the purpose of the systematic review.

Regardless of whether reviewers perform a meta-analysis or not, downgrading the quality of evidence for indirectness is appropriate when: (1) the final sample only represents a subset of the population of interest (an example of indirectness in population is displayed in Table 8); (2) when the complete breadth of the prognostic factor that is being considered in the review question is not well represented in the available studies (an example is illustrated in Table 8); or (3) when the outcome that is being considered in the review question is not broadly represented (an example is displayed in Table 8).

**Table 8:** The effect of considering indirectness when judging quality of evidence

**Factor**	**Example**
Indirectness in population	In our review we were interested in all headache sufferers. There were three longitudinal studies in the literature that examined the type of headache (that is, migraine or tension-type) as a prognostic factor for headache persistence [24,30,31]. All three studies recruited participants from headache clinics. These populations are not representative of all headache sufferers in the general population. It is likely that only those with more severe and frequent headache conditions are referred to a headache specialist [32]. We therefore downgraded the body of evidence on type of headache as a prognostic factor of headache persistence for serious indirectness.
Indirectness in prognostic factor	We cannot provide examples extracted from our review since our review was not intentionally limited to a specific prognostic factor. Instead our goal has been to explore all types of factors that have been investigated to date as potential risk or protective factors for the persistence of a variety of chronic pain conditions and their associated disability. However, this poor representation would happen, for example, if we were interested in exploring the effect of mental illnesses on the persistence of recurrent headaches and the primary studies included were only investigating the prognostic value of depression diagnosis on onset of headaches.
Indirectness in outcome	For example, this poor representation of outcome would happen if we were interested in exploring whether race is a risk factor for the persistence of recurrent headaches and the outcome was represented by studies assessing only the persistence of migraine (and at least not all types of the main primary headache disorders).

Downgrading the quality of the evidence with respect to indirectness depends on how extreme the differences are and how much these differences can influence the magnitude of effect. Reviewers should make this judgment based on the purpose of their review.

#### Imprecision

Random error or imprecision exists when the evidence is uncertain, leading to different interpretations about the relationship between the prognostic factor and its associated risk or protective value.

To judge whether the results of meta-analysis have sufficient precision a reviewer should first consider whether the number of participants included in the meta-analysis is appropriate through sample size estimation (similar to sample size estimates for a single study, but accounting for between-study heterogeneity; see Bull [33] for a discussion of the need of an adequate sample size in a meta-analysis). Second, if the number of participants included in the meta-analysis is appropriate, based on their best judgment, reviewers should consider the results precise when the confidence interval around the estimated effect size is not excessively wide while including values implying that the prognostic factor is associated with protection or increased risk.

Evaluating the imprecision when a meta-analysis is not possible is challenging. The best approach is for reviewers to judge the overall precision based on precision of results within each study while taking into account the number of studies and participants involved. If the majority of studies included in the review are precise, regardless of the number of studies and sample size, reviewers should not downgrade the quality of evidence for imprecision. To estimate whether the primary studies included in the review are unpowered or not, reviewers should take into account the sample size that each of the primary studies used. To do this, they should explore whether the authors of the primary studies provided any rationale for the sample size. Authors of prognostic studies should estimate an effect size and select the desired power beforehand, and then calculate the sample size needed in order to achieve that power and to detect the specified effect size [5,33]. If authors do not provide such rationale, reviewers can consider the sample size appropriate for studies using dichotomous outcomes using the 'rule of thumb’, when there are at least 10 outcome events for each potential prognostic variable considered in the analysis [4,34]. If insufficient information is available to determine appropriateness of sample size, or studies use continuous outcomes, the sample size can be considered appropriate when there are at least 100 cases that reached the endpoint [35]. If the sample sizes are large enough, the reviewers should then evaluate the width of the interval. If most of the confidence intervals reported in each study include both no effect and appreciable risk and protective values, the evidence derived from that particular study is imprecise. At that stage, to evaluate the overall imprecision for the explored prognostic factor association, we recommend reviewers to also consider the number of studies, and number of participants across studies, because there is likely to be more imprecision with a fewer number of studies and/or participants. Consequently, we recommend downgrading the quality of evidence if the evidence is generated by a few studies involving a small number of participants and most of the studies provide imprecise results. See an example in Table 9.

**Table 9:** The effect of considering imprecision when judging quality of evidence

**Factor**	**Example**
Imprecision	Menstruation in girls is another potential prognostic factor investigated in the pediatric headache literature. Wang and colleagues [22], the only study in our review that investigated this factor, sought to explore the prognostic value of menses in girls on migraine persistence in two follow-up annual surveys with a sample of 449 junior high school students with migraine. In their study, 134 out of 449 participants reported persistent migraine for all 3 years. Multiple logistic regression was used to explore the association between 14 potential prognostic factors (including menses in girls) and migraine persistence. This analysis has sufficient statistical power. No significant association between menses and persistent migraine was found; however, no effect size or confidence interval values were provided, so conclusions regarding the level of imprecision of the estimated effect of the menses remain unclear. Consequently, we considered the level of imprecision as unclear.

#### Publication bias

This is a very important factor in prognostic study evidence because investigators often fail to report relationships that show no effect between potential prognostic factors and outcomes. This happens when published evidence is restricted to only a portion of the studies conducted on the topic [36]. The current lack of an existing register for prognostic research studies prevents reviewers from making an informed judgment about whether there is evidence that publication bias is a potential problem. Consequently, a prudent default position at this moment is to assume that prognosis research is seriously affected by publication bias until there is evidence to the contrary [6]. Reviewers should consider that publication bias exists across all factors except in those cases in which they find that a determinate prognostic factor has been investigated in a large number of cohort studies. Ideally, most of these large numbers of studies should have been designed to purposefully confirm the hypothesized independent effect of the factor on the outcome (phase 2 study) or to test a conceptual model which explains its underlying mechanisms (phase 3 study). However, since phase of investigation is already taken into account as a factor that can downgrade the overall quality of evidence, we do not recommend downgrading again for publication bias due to only phase of investigation. For these cases, reviewers conducting a systematic review with or without meta-analysis may judge that there is less likely to be publication bias (see Table 10).

**Table 10:** The effect of considering publication bias when judging quality of evidence

**Factor**	**Example**
Publication bias	In our review, we identified research evaluating whether female (rather than male) sex can increase the risk for persistence of headache over time. Four longitudinal studies [28,37-39] at an early phase of investigation, explored this potential factor in multivariate analyses. All these studies explored the effect of female sex while including important potential confounders in the multivariate model. While Stanford and colleagues [38] reported female sex to be a significant risk factor when sex was entered simultaneously into the model with other co-variables (for example, anxiety, self-esteem, stressful life events), Monastero and colleagues [37], Termine and colleagues [39] and Wang and colleagues [28] reported no effect when adjusted for potential confounders such as age, medication overuse, age of onset, and psychiatric comorbidities. We considered this association explored by an adequate number of studies, and since we already downgraded for early phase of investigation we decided not to downgrade the quality of this evidence for publication bias.

### Reasons for rating up the quality of evidence

#### Moderate or large effect size

Multiple prognostic factors often contribute to the prognosis of health conditions. Therefore, finding a moderate or large effect size is one of the key criteria for rating up the quality of evidence. A moderate or large effect size increases the likelihood that a relationship between the prognostic factor and the outcome does in fact exist.

Reviewers should rate up the quality of evidence when they find a moderate or large pooled effect of the meta-analysis. 'Rules of thumb’ have been proposed to judge the effect moderate or large (for example, standardized mean difference statistic = around 0.5 for moderate effect or around 0.8 or larger for large effect [40], odds ratio = around 2.5 for moderate effect or 4.25 or greater for large effect [41,42]). However, because these are arbitrary guidelines, a sensible decision should be made and justified by the reviewers taking into account the study context (for example, background risk and unit of measurement).

If it is not possible to conduct a meta-analysis, reviewers should rate up the quality of evidence when they find moderate or large similar effects reported by most of the primary studies (see an example in Table 11).

**Table 11:** The effect of considering moderate or large effect size when judging quality of evidence

**Factor**	**Example**
Moderate or large effect size	For our review, we upgraded evidence for large or moderate effect sizes. For example, our review included two studies that examined through univariate analyses whether family history of headaches predicts migraine persistence. Monestero and colleagues [37] reported a significantly large effect (odds ratio = 6.2), while Ozge and colleagues [43] also examined the same relationship and reported a small-moderate effect size (odds ratio = 2.08). Collectively we felt these effect sizes were large enough to warrant upgrading the evidence, indicating that the relationship between family history of headache and persistence probably exists.

#### Exposure-response gradient

An exposure-response gradient exists when elevated levels of the prognostic factor (for example, larger amount, longer duration, higher intensity) lead to a larger effect size over lower levels of the factor. The presence of such a gradient increases confidence in the findings that the factor is associated with an increased risk or protective value and therefore raises our rating of the quality of evidence.

When conducting systematic reviews with meta-analysis, the reviewers should observe whether an exposure-response gradient is present between subgroup analyses for factors measured at different doses.

If a meta-analysis or subgroup analyses are not conducted, or only one meta-analysis is conducted for the relationship between a prognostic factor and outcome, reviewers should observe whether a possible exposure-response gradient consistently exists within and between primary studies. The use of the same measures to evaluate prognostic factors and outcomes across studies is a required condition to appropriately evaluate the possible existence of this gradient between studies. Table 12 displays an example.

**Table 12:** The effect of considering exposure gradient when judging quality of evidence

**Factor**	**Example**
Exposure gradient	In our review we did not observe evidence of an exposure-response gradient in any of the cases identified. However, an exposure-response gradient is observed in a cross-sectional study of headache, which was not included in our review because cross-sectional studies should not be used to study causes and prognosis. Anda and colleagues [44] evaluated, in a sample of adults with frequent headaches, whether the number of adverse childhood events (including childhood abuse and household dysfunction) prior to and including the age of 18 was associated with the presence of self-reported headaches in adulthood. A significant exposure-response gradient was identified, with an increase in the size of the odds ratio associated with the likelihood of headache in adulthood from 1.2, to 1.7, to 2.1 as the number of childhood adverse events also increases from 1, to 3, to 5, respectively.

The findings derived from the GRADE framework to judge the evidence derived from prognostic studies can be presented in the proposed summary of findings tables (see Tables 13 and 14).

**Table 13:** Example of an adapted Grading of Recommendations Assessment, Development and Evaluation (GRADE) table for systematic reviews with meta-analysis of prognostic studies

**Outcome:**													
					**GRADE factors**								
**Prognostic factors**	**Number of participants**	**Number of studies**	**Number of cohorts**	**Estimated effect size (95% confidence interval)**	**Phase**	**Study limitations**	**Inconsistency**	**Indirectness**	**Imprecision**	**Publication bias**	**Moderate/large effect size**	**Dose effect**	**Overall quality**


**Table 14:** Example of an adapted Grading of Recommendations Assessment, Development and Evaluation (GRADE) table for narrative systematic reviews of prognostic studies (filled in with examples of our own review illustrated in the boxes throughout this manuscript)

**Outcome: Headache persistence**																		
				**Univariate**			**Multivariate**				**GRADE factors**							
**Potential prognostic factors identified**	**Number of participants**	**Number of studies**	**Number of cohorts**	**+**	**0**	**-**	**+**	**0**	**-**	**Phase**	**Study limitations**	**Inconsistency**	**Indirectness**	**Imprecision**	**Publication bias**	**Moderate/large effect size**	**Dose effect**	**Overall quality**
Headache intensity	536	3 [22-24]	3	1	2	0				1	✕	✓	✓	✓	✕	✕	✕	+
Age	867	3 [26-28]	3	1	2	0				1	✕	✕	✓	✓	✕	✕	✕	+
Type of headache diagnosis	249	3 [24,30,31]	3	2	1	0				1	✕	✓	✕	✓	✕	✓	✕	+
Menstruation	449	1 [22]	1	0	1	0				1	✕	✕	✕	Unclear	✕	✕	✕	+
Sex	3,272	4 [28,38-40]	4	1	2	0	1	3	0	1	✕	✓	✓	✓	✓	✓	✕	+++
Family history of pain	654	2 [38,44]	2	2	0	0	1	1	0	1	✓	✕	✕	✓	✕	✓	✕	+

Phase, phase of investigation. For uni- and multivariate analyses: +, number of significant effects with a positive value; 0, number of non-significant effects; -, number of significant effects with a negative value. For GRADE factors: ✓, no serious limitations; ✕, serious limitations (or not present for moderate/large effect size, dose effect); unclear, unable to rate item based on available information. For overall quality of evidence: +, very low; ++, low; +++, moderate; ++++, high.

## Conclusions

This article is a first attempt to outline how GRADE may be adapted to assess the quality of evidence for prognostic research studies for a systematic review of the literature. To date, a formal system to guide researchers in assessing the evidence for prognostic studies has been lacking. Our adaptation is a first step in developing a systematic approach to evaluate the quality of evidence for prognostic research. We encourage these recommendations to be further developed and tested for GRADEing the quality of evidence when synthesizing findings from prognostic research studies. For instance, further guidance for reviewers is needed to decide when each of these GRADE factors causes the evidence to be down- or upgraded one versus two levels. At this stage, we leave this decision up to the judgment of the review teams to decide how much these factors impact the overall quality of evidence. Empirical research is also needed to explore our hypothesis that risk of bias detrimentally affects the factor-outcome relationship and what the strength of this relationship is.

## Abbreviations

GRADE: Grading of recommendations assessment, development and evaluation.

## Competing interests

The authors declare that they have no competing interests.

## Authors’ contributions

AH is the lead researcher of this project and wrote the first draft of the manuscript. AH, JAH, and MET formulated an initial proposal for the adaptation of the GRADE framework to prognosis research. JS, PJM, and CTC helped to reformulate this first proposal. AH, JAH, JS, PJM, CTC, MET and LW contributed significantly to the methodology, writing and revision of the manuscript. All authors read and approved the final manuscript.
